# Ultrasound-guided dual-localization for axillary nodes before and after neoadjuvant chemotherapy with clip and activated charcoal in breast cancer patients: a feasibility study

**DOI:** 10.1186/s12885-019-6095-1

**Published:** 2019-08-30

**Authors:** Won Hwa Kim, Hye Jung Kim, See Hyung Kim, Jin Hyang Jung, Ho Yong Park, Jeeyeon Lee, Wan Wook Kim, Ji Young Park, Yee Soo Chae, Soo Jung Lee

**Affiliations:** 1 Department of Radiology, School of Medicine, Kyungpook National University, Kyungpook National University Chilgok Hospital, 807, Hoguk-ro, Buk-gu, Daegu, 41404 Republic of Korea; 2 Department of Radiology, School of Medicine, Kyungpook National University, Kyungpook National University Hospital, 130, Dongdeok-ro, Jung-gu, Daegu, 41944 Republic of Korea; 3 Department of Surgery, School of Medicine, Kyungpook National University, Kyungpook National University Chilgok Hospital, 807, Hoguk-ro, Buk-gu, Daegu, 41404 Republic of Korea; 4 Department of Pathology, School of Medicine, Kyungpook National University, Kyungpook National University Chilgok Hospital, 807, Hoguk-ro, Buk-gu, Daegu, 41404 Republic of Korea; 5 Department of Oncology/Hematology, School of Medicine, Kyungpook National University, Kyungpook National University Chilgok Hospital, 807, Hoguk-ro, Buk-gu, Daegu, 41404 Republic of Korea

**Keywords:** Axillary nodes, Clipped node, Neoadjuvant chemotherapy, Localization, Neoadjuvant chemotherapy, Sentinel node, Tattooed node

## Abstract

**Background:**

We report on our experience of ultrasound (US)-guided dual-localization for axillary nodes before and after neoadjuvant chemotherapy (NAC) with clip and activated charcoal to guide axillary surgery in breast cancer patients.

**Methods:**

Between November 2017 and May 2018, a dual-localization procedure was performed under US guidance for the most suspicious axillary nodes noted at initial staging (before NAC, with clip) and restaging (after NAC, with activated charcoal) in 28 cytologically proven node-positive breast cancer patients. Patients underwent axillary sampling or dissection, which involved removing not only the sentinel nodes (SNs), but also clipped nodes (CNs) and tattooed nodes (TNs). Success (or failure) rates of biopsies of SNs, CNs, and TNs and inter-nodal concordance rates were determined. Sensitivities for the individual and combined biopsies were calculated.

**Results:**

SN biopsy failed in four patients (14%), whereas the CN biopsy failed in one patient (4%). All TNs were identified in the surgical field. Concordance rates were 79% for CNs–TNs, 63% for CNs–SNs, and 58% for TNs–SNs. Sensitivity for SN, CN, and TN biopsy was 73%, 67%, and 67%, respectively. Sensitivity was 80% for any combination of biopsies (SN plus CN, SN plus TN, SN plus CN plus TN).

**Conclusions:**

US-guided dual-localization of axillary nodes before and after NAC with clip and activated charcoal was a feasible approach that might facilitate more reliable nodal staging with less-invasive strategies in node-positive breast cancer patients.

## Background

Sentinel node (SN) biopsy is increasingly used in node-positive breast cancer patients undergoing neoadjuvant chemotherapy (NAC), as less-invasive surgical techniques for nodal staging have come to be more widely accepted for improving quality of life. In line with the findings of multiple trials, including ACOSOG Z1071 and SENTINA, the most recent American Society of Clinical Oncology guidelines state a moderate-strength recommendation for offering SN biopsy after NAC [1–3]. However, the false-negative rate (FNR) of SN biopsy may be higher than acceptable range (< 10%). In addition, identification rates of SNs have varied widely across studies (63–100%) [4]. Therefore, further strategies have been suggested to decrease the FNR. These include selection of patients with the greatest likelihood of having a complete response using ultrasound (US) and a modified SN biopsy approach, in which targeted nodes seen on US are removed along with the SNs.

Recently, several techniques using different materials have been used to localize targeted nodes [5]. For instance, nodes can be marked with radioactive iodine seeds placed at cytologically proven metastatic nodes before NAC [6]. Furthermore, targeted axillary dissection involves removing targeted nodes that have been marked with a metal clip before NAC and subsequently localized with radioactive iodine seeds after NAC [7, 8]. Tattooing with activated charcoal has also been used to localize targeted nodes before or after NAC; this approach has the benefits of convenience and being radiation-free, as well as being low cost [9]. Tattooing before NAC, however, does not allow tracking of the targeted nodes during NAC, because the activated charcoal cannot be seen on US.

Thus, we have developed a dual-localization technique in which a cytologically proven metastatic node is marked with a clip before NAC and tattooed with activated charcoal after NAC. Tattooing was also performed for the most suspicious node after NAC. This technique facilitates localization of targeted nodes at both initial staging and restaging, and evaluation of the inter-nodal relationships among the SN, the clipped node (CN), and the tattooed node (TN). Findings from our pilot study may assist in planning strategies to facilitate safer SN biopsy in node-positive breast cancer patients undergoing NAC. The goal of the present study was to report on our experience of US-guided dual-localization for axillary nodes before and after NAC with clip and activated charcoal to guide axillary surgery in breast cancer patients.

## Methods

### Patients

The institutional review board of our institution approved this prospective study. Between November 2017 and May 2018, 28 breast cancer patients with cytologically proven node-positive disease who were scheduled to undergo NAC agreed and signed informed consent for participation of this study. Fine-needle aspiration cytology was performed for the most suspicious nodes on US at initial staging. The NAC regimen generally included anthracycline-based treatment, consisting of doxorubicin and cyclophosphamide, followed by treatment with docetaxel. Patients with human epidermal growth factor receptor 2 (HER2) were additionally treated with trastuzumab.

### Dual-localization

**Fig. 1:**
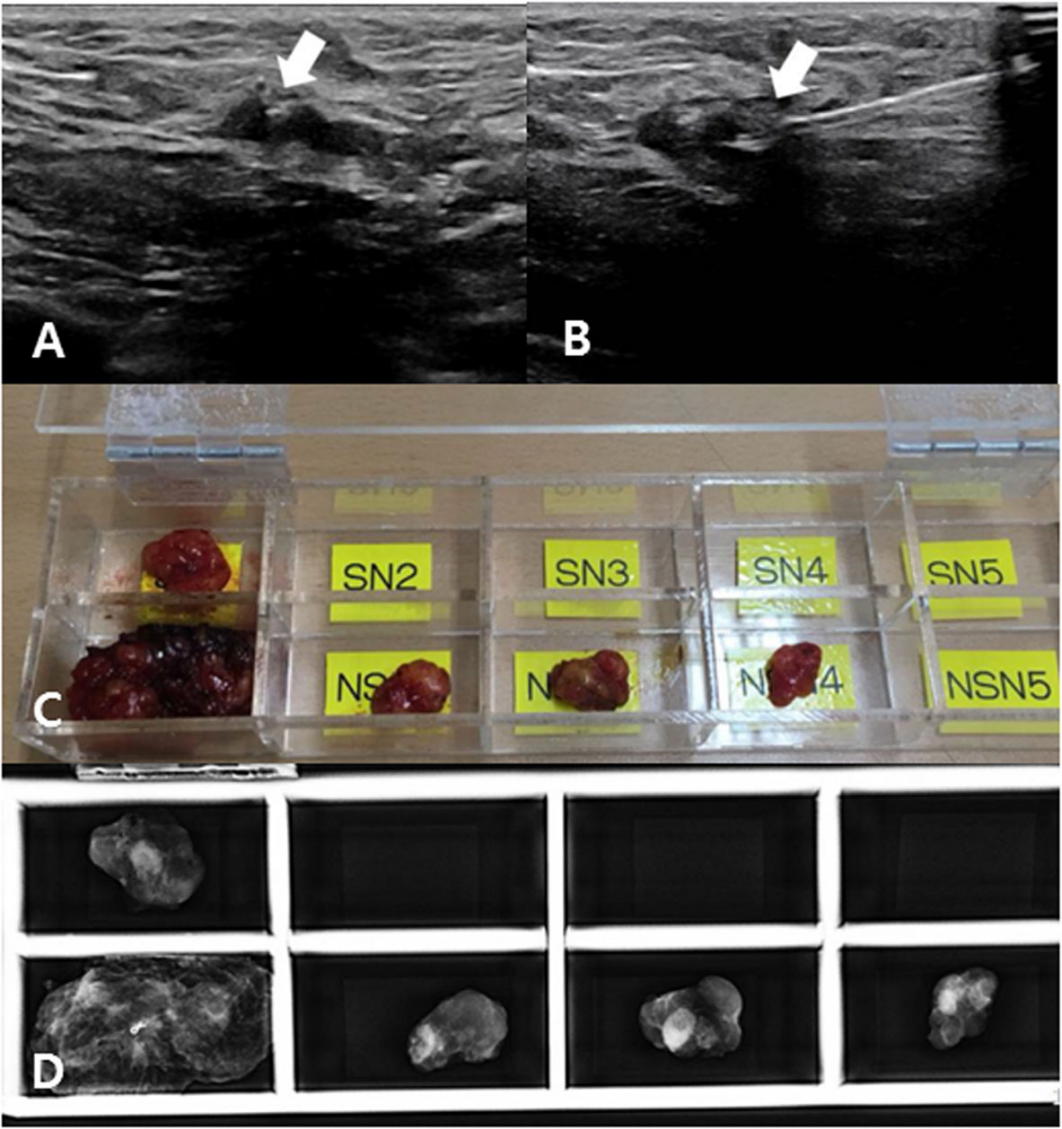
Ultrasonographic images at restaging after neoadjuvant chemotherapy (**a**, **b**) show the most suspicious axillary node, which had a clip (arrow, **a**) and was localized with activated charcoal (arrow, **b**). This tattooed node was a non-sentinel node (**c**) with a clip, identified in specimen mammography (**d**). Pathological results revealed metastases in both sentinel and tattooed nodes

Before commencing NAC, a metallic clip (ULTRACLIP® dual-trigger breast tissue marker, ultrasound-enhanced ribbon, BARD®, Tempe, AZ, USA) was placed on the cytologically proven metastatic nodes via a coaxial biopsy needle (TRUGUIDE®, BARD®, Tempe, AZ, USA) under US guidance after local anesthesia. CNs were followed-up on US during NAC (usually after four cycles of the NAC regimen). After completion of NAC (usually on the same day or 1 day before surgery), tattooing was performed for the nodes that appeared to be most suspicious on US at restaging. If the most suspicious node was not concordant with the CN, both the most suspicious node and the CN were tattooed. For tattooing, 1 ml of Charcotrace™ black ink (Phebra, Lane Cove West, Australia) was injected into the cortex of the node and adjacent soft tissue after local anesthesia (Fig. 1a, b). This procedure generally took approximately 5–20 min per patient. The radiologist marked location of the node on the skin with an oil-based pen to guide the surgical incision.

### Axillary surgery

After NAC, four attending breast surgeons determined surgical method and performed all the operative procedures. Although this study did not mandate a specific type of axillary surgery, targeted axillary sampling (TAS) was used as our standard protocol for node-positive breast cancer patients. TAS has been previously described [10, 11]. This technique involves not only removing (sampling) SNs (SN biopsy) but also TNs and several nodes around the SNs and TNs; this shared criteria was strictly applied by all surgeons during study period. The axillary vein, long thoracic nerve and thoraco-dorsal nerve were not exposed during TAS, whereas axillary dissection is defined as gross removal of most of the nodes with full exposure of those structures. SNs were identified with dual tracers (technetium-99 m phytate and blue dye) in all patients and defined as radioactive (technetium-99 m phytate) and/or blue dye-containing nodes. Blue dye-containing SNs were easily discriminated from TNs, because TNs have usually black charcoal ink in perinodal soft tissue with skin marking. If SNs could failed to be detected, sampling was performed under the guidance of TNs.

To evaluate the inter-nodal relationship among SNs, CNs, and TNs, all sampled nodes were placed in a pre-designed acrylic box with multiple slots (Fig. 1c). SNs were placed in the SN-slots and named in order of higher level of radioactivity (SN1, SN2 …). Non-SNs (nodes without radioactivity or blue dye) were placed in the non-SN slots (NSN) and named (NSN1, NSN2 …). Specimen mammography was taken for the nodes in the acrylic box and radiologists identified and recorded which nodes were CNs or TNs (Fig. 1d). Then, the radiologists placed a pin in the clip and submitted the sampled nodes for producing frozen sections intraoperatively. If the pathological result of the frozen sections revealed metastases, axillary dissection was usually performed.

### Pathological evaluation

For intraoperative frozen sections, the nodes were bisected, and a single 5-μm-thick section was stained with hematoxylin and eosin. After obtaining a frozen section, the nodes were fixed in formalin, embedded in paraffin, and sectioned for routine hematoxylin–eosin staining. Each node was finally classified as negative or positive for metastases, and the numbers of nodes that were resected and that had metastases were recorded.

### Statistical analysis

The clinical data collected included age at cancer diagnosis, menopausal status, clinical T stage, clinical N stage, and number of suspicious nodes on US at initial staging and restaging. The definition of suspicious nodes was based on previous studies [12–15]. The following histopathological information was included in the study: histological tumor characteristics, nuclear grade, histological grade, estrogen receptor (ER), progesterone receptor (PR), and HER2 status. Tumors expressing ER and/or PR were defined as hormone receptor (HR)-positive. A HER2 score of 0 or 1 was considered HER2-negative, a value of 3 was considered HER2-positive, and a value of 2 was considered equivocal. For equivocal cases, silver-enhanced in situ hybridization was performed, and a HER2/CEP17 ratio of ≥2 or HER2/CEP17 ratio of < 2 with an average HER2 copy number of ≥6 were considered HER2-positive [16].

The primary outcome was the success (or failure) rate of identifying SNs, CNs, and TNs as well as their inter-nodal relationship. Outcomes according to clinical N stages and the number of retrieved SNs were compared using the chi-square test for trend and Fisher’s exact test, as appropriate. The sensitivity of the individual or combined biopsies was the secondary outcome. All statistical analyses were performed using MedCalc v.17.1 (Mariakerke, Belgium).

## Results

The clinicopathological details of the 28 patients (mean age, 49 years; range, 30–67 years) are described in Table 1. Nineteen patients (68%) had cN1, five patients (18%) had cN2, and four patients (14%) had cN3. The median number of suspicious nodes on US at initial staging was three (range, 1–11). At restaging US, five (18%) patients had suspicious nodes (one node in four patients and three nodes in one patient) and 23 patients (82%) had no suspicious nodes. Among these, six clips (21%) were equivocally visible and 22 clips (79%) were clearly visible at restaging US. Twenty patients (71%) underwent TAS and eight patients (29%) underwent axillary dissection. The median number of resected nodes was seven (range, 2–22); five (range, 2–14) in TAS and 15 (range, 8–22) in axillary dissection. On final pathological reports, 13 patients (46%) had no metastatic nodes (ypN0), while 15 patients (54%) had metastatic nodes with ypN1 in 11 patients (39%), ypN2 in one patient (4%), and ypN3 in three patients (11%).

**Table 1:** Clinicopathological features of the patients

Characteristics	Number of patients
Menopausal status	
Premenopausal	20 (71%)
Postmenopausal	8 (29%)
Clinical T stage	
T1	3 (11%)
T2	19 (68%)
T3	3 (11%)
T4	3 (11%)
Clinical N stage	
N1	19 (68%)
N2	5 (18%)
N3	4 (14%)
Histologic tumor characteristic	
Ductal	26 (93%)
Ductal vs. lobular	2 (7%)
Nuclear grade	
Low	0
Moderate	13 (46%)
High	15 (54%)
Histologic grade^a^	
Low	3 (11%)
Intermediate	11 (39%)
High	6 (21%)
Missing	8 (29%)
HR status	
Negative	11 (39%)
Positive	17 (61%)
HER2 status	
Negative	20 (71%)
Positive	8 (29%)

*HR* hormone receptor, *HER2* human epidermal growth factor receptor 2

^a^Modified Scarff–Bloom–Richardson grading system

SN biopsy failed in four patients (14%) because of failure to detect the SN, despite faint radioisotope uptake on lymphoscintigraphy. The SN biopsy failure rate tended to increase with higher clinical N stage (0% [0/19] in cN1, 20% [1/5] in cN2, and 75% [3/4] in cN3; *P* < .001). There was one SN in 11 patients (46%; nine in cN1, one in cN2, and one in cN3), two in 10 patients (42%), and three in three patients (13%). CN biopsy failed in one patient (4%) with cN2; when the radiologist tattooed the most suspicious node that appeared to have a clip at restaging. The patient’s postoperative mammography showed the clip in the axilla; clip dislodgement was not seen on the latest follow-up. All TNs were identified in the surgical field. The success rate (100%) of TN biopsy was significantly higher than that of SN biopsy (86%, *P* = .004).

The concordance rate between CNs and TNs was 79% (22/28), suggesting a discordance rate of 21% (6/28) between initial staging and restaging in US assessments of nodes mostly likely to have metastases. The concordance rate between CNs and SNs and between TNs and SNs was 63% (15/24) and 58% (14/24), respectively. The discordance rate between CNs and SNs and between TNs and SN was 38% (9/24) and 42% (10/24), respectively, indicating that substantial disagreement was observed in the SNs and US-assessed suspicious nodes at initial staging or restaging.

The inter-nodal relationships according to the clinical N stages or the number of retrieved SNs are described in Tables 2 and 3. Discordance rates were generally higher in groups with higher clinical N stages or with one retrieved SN than in groups with lower clinical N stages or with two more retrieved SNs; however this did not reach a statistical significance. Of 19 patients with cN1, 10 patients had metastatic nodes; in these patients, all SNs (sensitivity, 100%) and eight CNs (concordant with TNs, sensitivity 80%) showed metastases. Of five patients with cN2, three patients had metastases; one SN (sensitivity, 33%) and two CNs (concordant with TNs, sensitivity, 67%) showed metastases. Of four patients with cN3, two patients had metastases; in these patients, none of the SNs, CNs, or TNs showed metastases (all sensitivity, 0%).

**Table 2:** Inter-nodal relationships according to clinical N stage

Relationship	All	cN1	cN2	cN3	*P* value
Clipped node to tattooed node					.621
Concordance	79% (22/28)	79% (15/19)	60% (3/5)	100% (4/4)	
Discordance	21% (6/28)	21% (4/19)	40% (2/5)	0% (0/4)	
Clipped node to sentinel node^a^					.156
Concordance	63% (15/24)	68% (13/19)	50% (2/4)	0% (0/1)	
Discordance	38% (9/24)	32% (6/19)	50% (2/4)	100% (1/1)	
Tattooed node to sentinel node^a^					.691
Concordance	58% (14/24)	58% (11/19)	75% (3/4)	0% (0/1)	
Discordance	42% (10/24)	42% (8/19)	25% (1/4)	100% (1/1)	

^a^Sentinel nodes were narrowly defined as radioactive nodes and/or nodes containing blue dye

**Table 3:** Inter-nodal relationships according to the number of retrieved sentinel nodes (SNs)

Relationship	One SN	Two or more SNs	*P* value
Clipped node to tattooed node			1.00
Concordance	73% (8/11)	77% (10/13)	
Discordance	27% (3/11)	23% (3/13)	
Clipped node to sentinel node^a^			.206
Concordance	45% (5/11)	77% (10/13)	
Discordance	55% (6/11)	23% (3/13)	
Tattooed node to sentinel node^a^			.102
Concordance	36% (4/11)	77% (10/13)	
Discordance	64% (7/11)	23% (3/13)	

^a^Sentinel nodes were narrowly defined as radioactive nodes and/or nodes containing blue dye

Overall, the sensitivity for SN, CN, and TN biopsy was 73% (11/15), 67% (10/15), and 67% (10/15), respectively. The sensitivity for any combination of biopsies was 80% (12/15), which was higher than that of the individual biopsies. Sensitivities differed significantly according to clinical N stages (Table 4).

**Table 4:** Sensitivities of sentinel, clipped, and tattooed node biopsy

Sensitivity of Node Biopsy	All	cN1	cN2	cN3	*P* value
Sentinel^a^	73% (11/15)	100% (10/10)	33% (1/3)	0% (0/2)	<.001
Clipped	67% (10/15)	80% (8/10)	67% (2/3)	0% (0/2)	.042
Tattooed	67% (10/15)	80% (8/10)	67% (2/3)	0% (0/2)	.042
Sentinel plus Clipped	80% (12/15)	100% (10/10)	67% (2/3)	0% (0/2)	.001
Sentinel plus Tattooed	80% (12/15)	100% (10/10)	67% (2/3)	0% (0/2)	.001
Sentinel plus Clipped plus Tattooed	80% (12/15)	100% (10/10)	67% (2/3)	0% (0/2)	.001

^a^Sentinel nodes were narrowly defined as radioactive nodes and/or nodes containing blue dye

## Discussion

With advances in NAC for breast cancer patients with cytologically proven node-positive disease, the eradication rate of nodal metastases now is approximately 40–75% after NAC [17–19]. This substantial rate has prompted less-invasive strategies for surgical nodal staging. To date, most strategies have involved removing SNs and/or targeted nodes, which are often localized by means of a clip. The National Cancer Comprehensive Network guidelines recommend clip placement before NAC, because CN biopsy along with SN biopsy reduces the FNR [20]. However, invisibility of CNs during surgery needs further localization technique with materials of iodine seed or wire [21]. Iodine seeds have been suggested by studies in the US and Netherlands, but they are not available in many other countries. Use of such seeds also requires a special device, with the accompanying regulations of handling and disposal of radioactive materials. The wire has also been used for localizing axillary nodes in some prospective studies [14, 21, 22]. It induces pain and discomfort in patients prior to their surgical removal. Activated charcoal, as suggested in this study, is a safe, convenient, and cheap option for localizing CN [23–25]. In addition, we obtained a perfect identification rate for TNs, which indicates that TN biopsy is an uncomplicated approach for surgeons. Tattooing with activated charcoal has been reported to yield high identification rates in previous studies [9, 11, 26]. Two studies involved tattooing after NAC [9, 11] while another study involved tattooing before NAC [26]. The strength of our study is that we performed tattooing after NAC for the nodes clipped before NAC, allowing us to evaluate the inter-nodal relationship as well as the technical feasibility of the approach.

We found considerable discordance between SNs and US-guided targeted nodes (CNs or TNs), and between CNs and TNs. Discordances rates tended to increase with higher clinical N stages overall, although this did not reach a statistical significance, given the small number of patients. Discordance between CNs and TNs suggests the disagreement in assessments for nodal status at initial staging and restaging. In particular, it is not easy that choosing only one suspicious node that appeared to be the most suspicious at initial staging, because node-positive patients may have multiple nodes showing aggregation and perinodal inflammation. Variability of chemotherapy response among nodes (intratumoral heterogeneity) may limit the initial staging-based nodal sampling. Therefore, restaging may play a role in predicting nodal status. Imaging (usually US) has been recommended for guiding axillary surgery in previous studies, despite the moderate sensitivity of this approach [13, 27, 28].

Discordance between SNs and US-guided targeted nodes (CNs or TNs) suggests that SN biopsy may yield false-negatives. In addition, despite this substantial discordance, the overall sensitivity for SN, CN, and TN biopsy was similar. The highest sensitivity was achieved using any combination of SN and targeted node (CN or TN) biopsy. Our findings demonstrate the potential role of sampling of US-guided targeted nodes noted at initial staging or restaging, along with SN biopsy in node-positive breast cancer patients undergoing NAC. However, further studies are required to determine the role of our dual-localization technique for reducing the FNR of SN biopsy to below an acceptable level, with a greater number of patients and using complete axillary dissection.

In this study, the failure rate of SN biopsy was 14%, and only one SN was identified in 46% patients. In the ACOSOG Z1071 and NSABP B-27 trials, the SN could not be identified in 7% and 15% of patients, respectively; only one SN was excised in 12% and 41% of patients in these trials, respectively. We found that the SN biopsy failure rate tended to increase with higher clinical N stage (*P* < .001). In our previous study, a similar finding was observed: 3% (1/29) in the cN1 group vs. 25% (4/16) in the cN2 or higher group [11]. In some of previous studies, FNRs were also higher in patients with higher clinical N stages [7, 29, 30] This low SN identification rate and possibly high FNRs in patients with higher clinical N stages may be associated with chemotherapy-induced fibrosis in the lymphatic channel [31]. A higher tumor burden in the lymphatics may result in more fibrosis, raising the possibility of lymphatic channel obstruction. However, this association has not been elucidated in previous studies [2, 32]. Other previous studies showed no significant correlation of the SN identification rate or FNR with clinical N stage [1, 32], possibly for the following reasons: 1) A wide spectrum of definitions of SNs [33, 34]: some studies have included palpable nodes in the surgical field as SNs and other studies did not; 2) variability in clinical N staging: our nodal staging system is mainly based on US findings (quantified by the number of suspicious nodes) at initial staging, as compared to physical examination and/or US findings that are used in many institutions.

We faced a challenge in US-guided dual-localization technique suggested in this study. Although clips were easily placed in all cases, without significant complications, 21% of clips were not clearly visible on US performed after NAC, as demonstrated previously in several studies [21, 35]. Although the hyperechoic (metallic) clip is easily visible against the background hypoechoic cortex of the axillary node before NAC, the cortex becomes thinner as NAC proceeds, which hinders differentiating the clip from echogenic fat strands. Thus, using a different type of clip that is easily visible on US can be considered as an approach for tagging targeted nodes.

This study had several limitations. The number of patients for this pilot study is relatively small. To confirm node-positive disease at initial staging, fine-needle aspiration cytology was employed rather than core-needle biopsy; hence, whether the nodal deposits are macrometastases or micrometastases are unknown. Further investigations in larger populations possibly with core-needle biopsy for axillary nodes are needed to confirm our findings and provide greater understanding of the clinical implications.

## Conclusion

Our study found that US-guided dual-localization of axillary nodes before and after NAC with clip and activated charcoal was a feasible approach that might facilitate more reliable nodal staging, with less-invasive strategies in node-positive breast cancer patients.

## Data Availability

The dataset used and/or analysed during the current study are available from the corresponding author on reasonable request.

## Abbreviations

CN: Clipped node
ER: Estrogen receptor
FNR: False-negative rate
HER2: Human epidermal growth factor receptor 2
HR: Hormone receptor
NAC: Neoadjuvant chemotherapy
NSN: Non-sentinel node
PR: Progesterone receptor
SN: Sentinel node
TAS: Targeted axillary sampling
TN: Tattooed node
US: Ultrasound
